# Unexpected dislocation following accurate total hip arthroplasty caused by excessive hip joint laxity during myasthenic crisis: a case report

**DOI:** 10.1186/s13256-018-1886-6

**Published:** 2018-11-06

**Authors:** Yoshiki Murotani, Yutaka Kuroda, Koji Goto, Toshiyuki Kawai, Shuichi Matsuda

**Affiliations:** Department of Orthopaedic Surgery, Graduate School of Medicine, Kyoto University, Shogoin, Kawahara-cho 54, Sakyo-ku, Kyoto, 606-8507 Japan

**Keywords:** Myasthenia gravis, Total hip arthroplasty, Dislocation, Osteonecrosis, Combined anteversion

## Abstract

**Background:**

Dislocation following total hip arthroplasty is mainly caused by malposition. However, the coexistence of neuromuscular disorders is also considered a risk for dislocation due to excessive hip joint laxity. To minimize risk of dislocation, preoperative planning using combined anteversion has been widely used. The recommended combined anteversion angle (the total of cup and stem anteversion angles) is 50 ± 10°.

**Case presentation:**

A 33-year-old Japanese woman underwent elective total hip arthroplasty due to osteonecrosis of the femoral head associated with corticosteroid pulse therapy for myasthenia gravis. Intraoperatively, no tendency of dislocation was found when simulating an evoking position under general anesthesia. In postoperative X-ray and computed tomography scans, cup inclination, cup anteversion, and stem anteversion angles were 37°, 13°, and 35° respectively. The resulting combined anteversion was 48°, which was set as the target along with accurate placement. Her postoperative course was normal and she was discharged without adverse events. Three months postoperatively, due to worsening of myasthenic weakness in her lower extremities while resting, she tended to raise her left limb up using both hands for sitting up. An anterior dislocation occurred when her legs were in a figure-of-four position. She was brought to an emergency department, and reduction of dislocation was performed. It was inferred that myasthenic crisis in the affected limb enabled excessive passive motion due to joint hyperlaxity.

At the end of 2016, elective total hip arthroplasty on the contralateral side was performed. Cup anteversion, stem anteversion, and the combined anteversion angles were 27°, 24°, and 51° respectively. We instructed her to exercise care during passive leg movement, which may worsen her myasthenic condition. She returned to a normal life and was able to walk long distances without a cane. No recurrence of dislocation was seen at final follow-up.

**Conclusions:**

Even if accurate component orientation is attained in total hip arthroplasty, patients with neuromuscular disorders such as myasthenia gravis have a potential risk of muscle weakness in the affected limb. Therefore, physicians’ instructions and patients’ careful attention are required to prevent dislocation due to excessive hip joint laxity under conditions of motor weakness.

## Background

Total hip arthroplasty (THA) is considered the definitive treatment for patients with osteonecrosis of the femoral head (ONFH). Although the etiology of ONFH remains unexplained, corticosteroid therapy is recognized to be the most causative factor for ONFH. Therefore, many diseases needing corticosteroid therapy, including myasthenia gravis (MG), have a potential risk of steroid-induced ONFH. MG is an uncommon disorder (200–400 cases per million). It is an autoimmune disease caused by abnormal neuromuscular transmission. The symptoms usually progress from extrinsic ocular muscles to other bulbar muscles, muscles in the extremity, and respiratory muscles [1, 2]. In the course of worsening MG, patients experience difficulty during normal muscular movements such as standing up without help. This leads to a significantly different range of motion in these patients compared with individuals without MG. Several treatments for MG have been developed, anti-cholinesterase therapy being the most common. However, in the case of anti-cholinesterase therapy failure, corticosteroid therapy or thymectomy are the next most commonly used approaches. In recent years, regardless of MG status, patients who also present with thymoma are advised to undergo resection [3]. Treatment of myasthenic crisis has been performed using systemic steroid pulse therapy [1–3]. However, systemic corticosteroid pulse therapy may increase risk of adverse events including ONFH. ONFH is also a rare condition but may cause femoral head collapse and disability in walking. Even in young patients, THA is needed to treat arthrosis, the final stage of ONFH. Although THA is one of the most successful surgical procedures in orthopedics, complications involving longevity, infection, or dislocation still exist. Dislocation following THA remains one of the serious complications, which is mainly caused by impingement problems including malposition, contact between bony pelvis and bony femur, and hyperlaxity [4–6]. To minimize risk of postoperative dislocation, the Lewinnek safe zone for cup placement (inclination of 40° ± 10° and anteversion of 15° ± 10°) has been considered a gold standard in acetabular component orientation [7]. Recently, combined anteversion (CA) has been proposed, developed, and widely used as an advanced approach [8, 9]. The CA angle, the sum of cup and anteversion angles, should be 50 ± 10° [10] because it has been reported that dislocation risk is 6.9 times higher if the CA angle was outside the range of 40°–60° [11]. Although several studies have reported the use of THA using CA avoiding malposition or impingement [10–12], there are few reports of dislocation following THA caused by hyperlaxity. Coexistence of neuromuscular disorders such as MG is considered one of the risk factors for dislocation due to weakness of the hip joint. Here we describe a rare case of unexpected dislocation after accurate alignment in THA due to excessive hip joint laxity during myasthenic crisis.

## Case presentation

In the summer of 2007, a 25-year-old Japanese woman (height 161 cm, body weight 80 kg, body mass index 30.8 kg/m^2^) felt weakness in both upper limbs and could not raise both arms while washing her face. Suspected neuromuscular symptoms included eyelid ptosis, trismus when gargling, and inability to stand due to weakness in lower extremities. She was immediately hospitalized in the internal medicine department at Kyoto University for further evaluation. No particular family and personal medical history were reported. Bilateral eyelid ptosis, masticatory muscle fatigue, and diffuse weakness of all limbs, denoting Osserman IIA classification, were noted on physical examination. A Tensilon test was positive, and the serum acetylcholine receptor antibody (anti-AChR) level was markedly elevated (32 nmol/L), which was strongly indicative of MG. Electromyography showed a waning phenomenon. In addition, a computed tomography (CT) scan revealed a thymoma. After evaluating these clinical findings, she was diagnosed as having MG. According to the Myasthenia Gravis Foundation of America (MGFA) scale, her muscular weakness was categorized as class IIIa. At the time of initial diagnosis, in July 2007, she did not prefer surgical resection of the thymoma, and received orally administered anti-cholinesterase inhibitor (pyridostigmine 60 mg/day). However, her symptoms did not improve. She was therefore given a corticosteroid (oral methylprednisolone up to 30 mg/day) in addition to the anti-cholinesterase inhibitor. Although symptoms of MG were initially relieved, at the beginning of the year 2010, clinical recurrence of MG was observed. The serum anti-AChR level was markedly elevated at 46 nmol/L. She was readmitted to our hospital with weakness of the extremities and worsened ptosis. She received systemic steroid pulse therapy, and the first course comprised 1000 mg of methylprednisolone administered intravenously for 3 days. Finally, in April 2010, she underwent thymectomy performed via a trans-sternal approach using video-assisted thoracoscopic surgery. During the perioperative period, she received intravenous corticosteroid pulse therapy three times, amounting to a total of 12,000 mg methylprednisolone equivalent. Myasthenic weakness improved MGFA IIa; administration of corticosteroid was gradually tapered to 20 mg/day as a maintenance dose. Her serum anti-AChR level was decreased to 11 nmol/L.

**Fig. 1:**
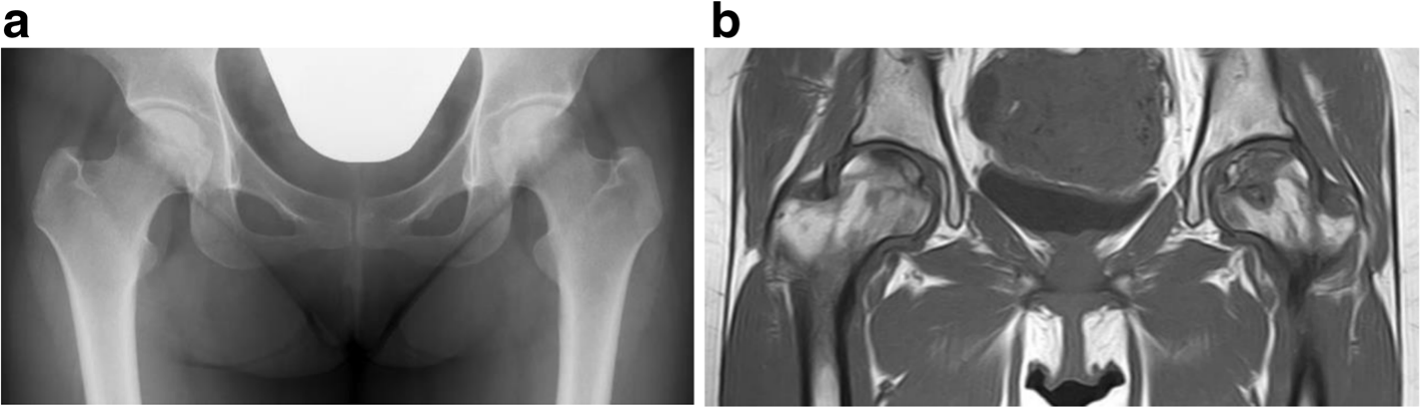
Anteroposterior radiograph of both hips (**a**) and T1-weighted magnetic resonance coronal image of both hips (**b**) showing bilateral osteonecrosis of the femoral head; stage 3A/3A, type C1/C2

**Fig. 2:**
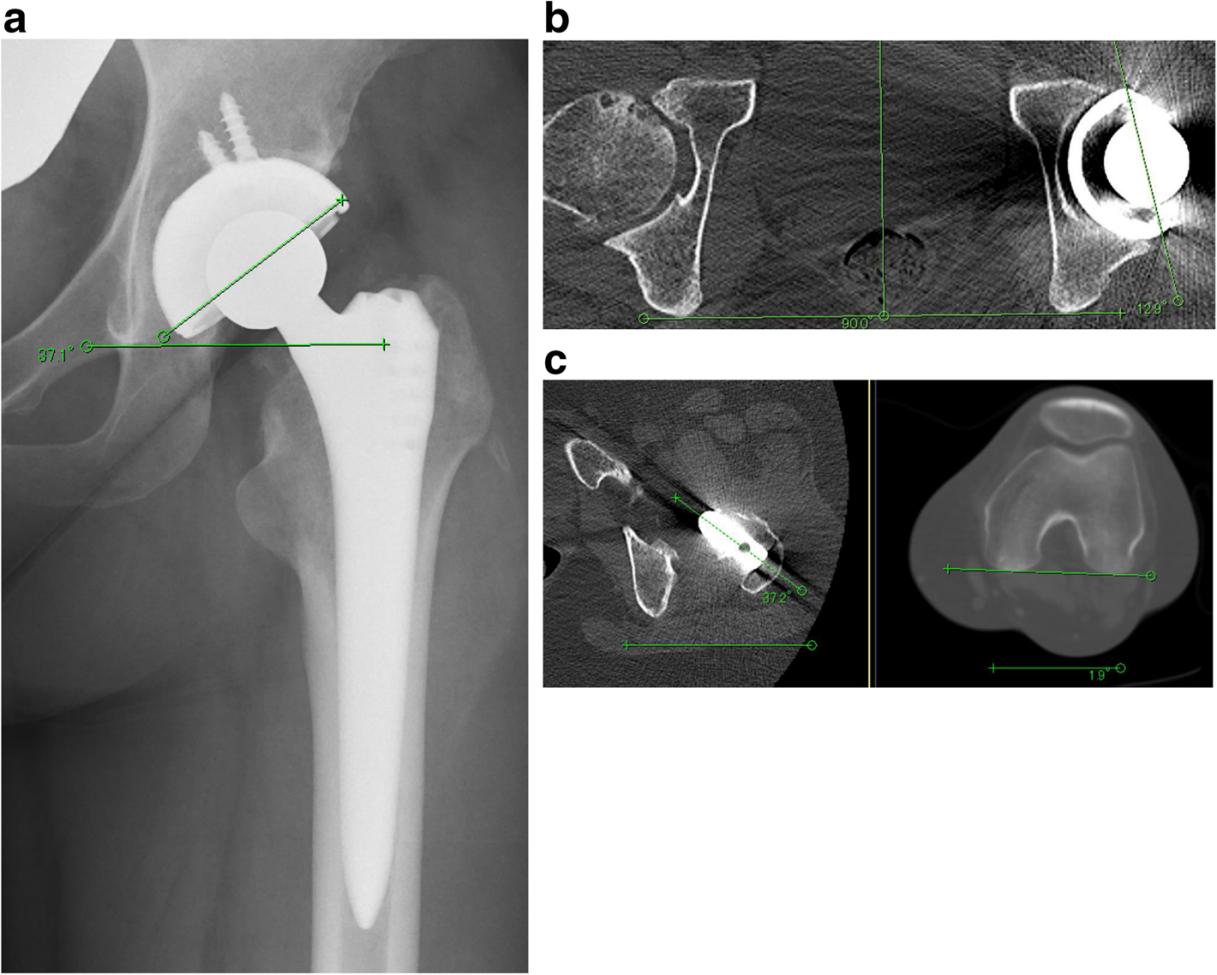
Postoperative radiograph (**a**) and postoperative tomography (**b**, **c**) showing cup inclination 37°, cup anteversion 13°, and stem anteversion 35°. Although the stem anteversion was slightly high, the total anteversion of the cup and stem was 48° which was within the targeted range of 50 ± 10° anteversion needed to avoid dislocation following total hip arthroplasty using combined anteversion

At the end of the year 2014, when she was 32-years old, sudden right hip arthritis occurred. She was referred to our orthopedic department during the next 2 months. We suspected a case of steroid-induced ONFH because she received repeated systemic corticosteroid pulse therapy for MG. There were abnormal signs in magnetic resonance imaging of her bilateral hip joints. Thus, she was diagnosed as having bilateral stage 3A, type C (type C1 in right hip, type C2 in left hip) ONFH (Fig. 1) classified based on the 2001 revised Japanese Investigation Committee guidelines [13]. After bilateral ONFH diagnosis, she complained of continuous and hip joint pain on both sides, stronger on the left than on the right. Therefore, she was scheduled for a left primary THA initially. In October 2015, an elective left THA was performed via an anterolateral approach with our patient in a lateral position using the cementless THA system (R3 acetabular cup, SL-PLUS stem, and 28 mm Oxinium head on highly cross-linked polyethylene; Smith & Nephew). The cementless cup was first placed targeting 20° anteversion using the manufacturer’s cup inserter and 40° abduction by aligning the jig to the longitudinal axis of the body. Using the CA technique, stem anteversion was coordinated with cup anteversion targeting a CA angle of 50 ± 10°. During surgery, we confirmed that there was no tendency of dislocation by simulating dislocation of the hip under general anesthesia (maximum flexion with maximum internal rotation for simulating posterior dislocation, and maximum external rotation with maximum extension for simulating anterior dislocation). We confirmed proper alignment of the prosthesis by evaluating postoperative radiographs. In the analysis of postoperative X-ray and CT scans after left THA, the cup inclination angle, the cup anteversion angle, and the stem anteversion angle were 37°, 13°, and 35°, respectively. The resulting CA angle was 48° (Fig. 2). Her postoperative course was normal, and following rehabilitation therapy including conventional training of daily living, standing up from the floor, sitting straight, and sitting cross legged, she became ambulatory and was discharged.

**Fig. 3:**
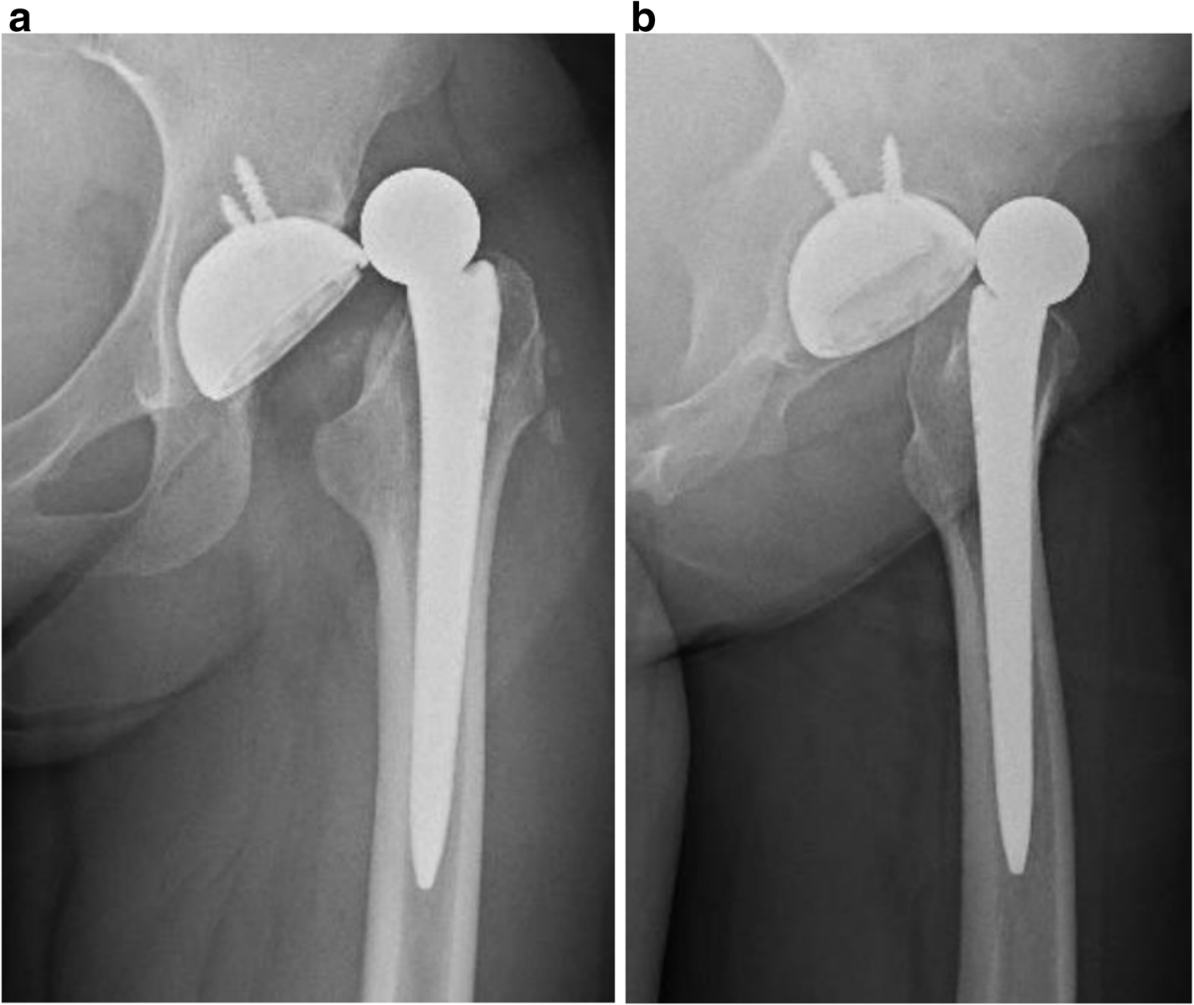
Anteroposterior radiograph (**a**) and frog leg view (**b**) showing anterior dislocation of the left hip prosthesis

**Fig. 4:**
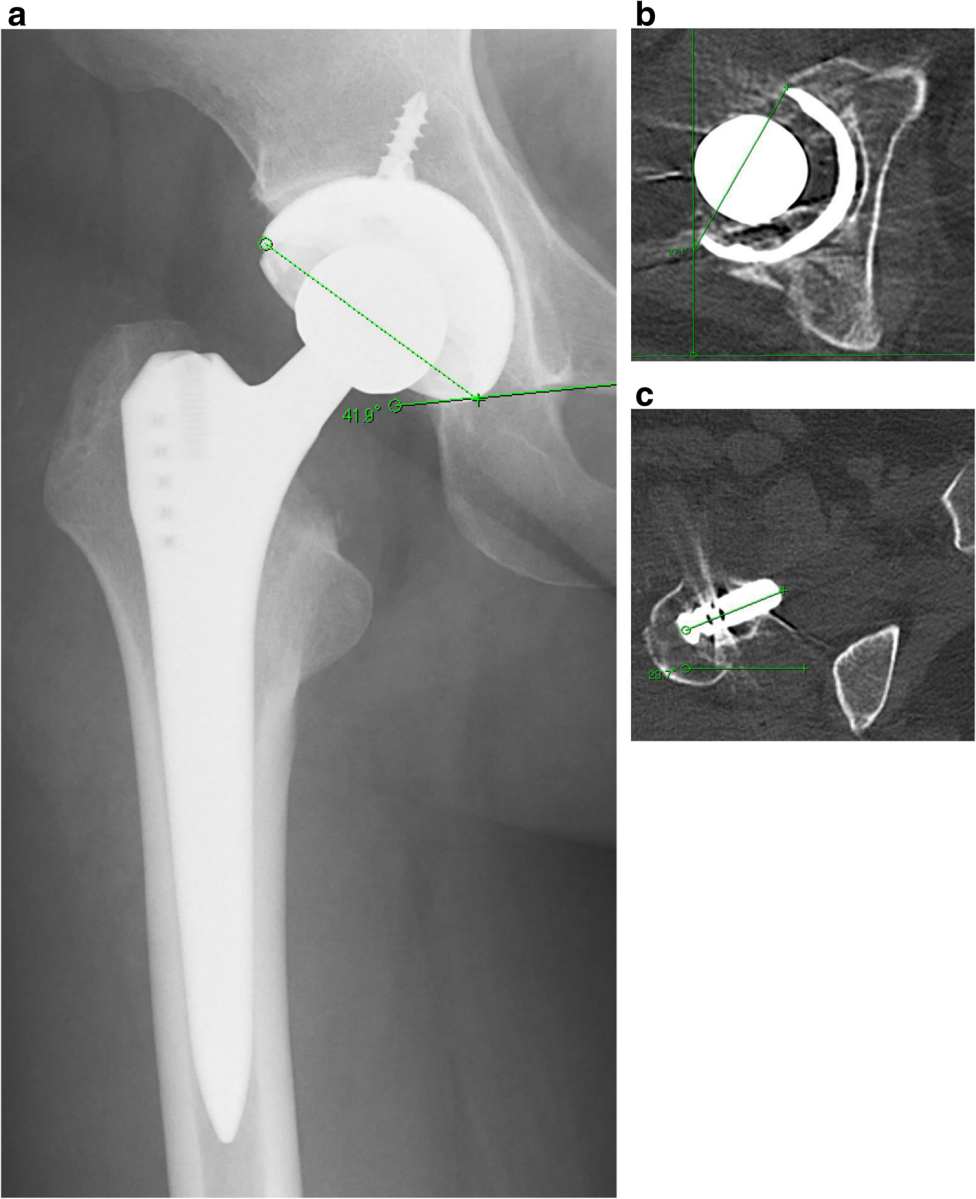
Radiological assessments of contralateral side total hip arthroplasty. Postoperative radiograph (**a**) and postoperative tomography (**b**, **c**) showing cup inclination 42°, cup anteversion 27°, stem anteversion 24°, and combined anteversion 51°

Three months after surgery, due to sudden occurrence of myasthenic weakness in her lower extremities while resting on a bed, she tended to raise her left limb up using both hands for sitting up. With her left leg in a figure-of-four position, she experienced sudden-onset severe pain and locking of left hip movement. She was diagnosed as having anterior dislocation following THA (Fig. 3) and treated with closed reduction under sedation at the emergency department of another hospital. One year postoperatively, her University of California, Los Angeles (UCLA) activity score for her left hip improved to 4 from a preoperative activity score of 3 and her Harris Hip Score (HHS) improved to 81.0 points in her left hip compared with a preoperative HHS of 49.0 points. She was followed up as an out-patient but right hip pain developed gradually. At the end of the year 2016, an elective right THA was performed as was done earlier on her left side. Postoperative radiographic assessment using CT images after right THA showed that the cup inclination angle, the cup anteversion angle, the stem anteversion angle, and the CA was 42°, 27°, 24°, and 51° respectively (Fig. 4). No postoperative complications were observed. We instructed her to exercise care during passive movements of her left leg, which may worsen her MG condition. No recurrence of dislocation in either hip has been observed since. Clinical scores in her right hip improved (UCLA activity score, 5; and HHS, 81.0 points) compared with those preoperatively (UCLA activity score, 4; HHS, 65.0 points) at the final follow-up. She returned to a normal life and has been able to walk long distances without a cane. We are carefully following her up as an out-patient.

## Discussion

We encountered a patient with unexpected dislocation following an elective THA caused by excessive passive motion because of joint hyperlaxity due to myasthenic crisis. MG is an autoimmune syndrome caused by the failure of neuromuscular transmission resulting from binding of autoantibodies to proteins involved in signaling at the neuromuscular junction [1, 2]. In the course of worsening MG, fatigability, muscle weakness, and joint laxity typically present. Muscle weakness in a limb which has received THA leads to excessive passive motion due to hip joint hyperlaxity, possibly causing dislocation due to movement beyond the general range of motion. With the prevalence of preoperative planning using CT images, CA has been established as a modern technique to avoid dislocation caused by malposition or impingement [8–12]. The CA technique has been preferred in patients with femoral anteversion such as that seen in dysplastic hips as well as normal hips [9, 10, 14]. To achieve an accurate CA, we use the CA technique following Dorr’s recommended [12] method of adjusting cup anteversion based on stem anteversion in cementless THA, with the targeted range of CA from 40° to 60° to avoid dislocation and maximize range of motion in the hip [10, 11].

We confirmed accurate placement of the prosthesis by evaluating postoperative X-ray radiographs and CT images, and the resulting CA was 48°, almost ideal using the CA technique. Therefore, we surmised that the cause of dislocation was excessive passive motion of the affected limb due to hyperlaxity during myasthenic crisis. We explained the potential risk of dislocation due to worsening MG to our patient, and instructed her not to move the affected foot passively. She experienced no further left hip dislocation during the 2 years since then, and has functioned without a hip abduction device.

Accurate component placement has been considered essential in successful THA. Several studies have reported risk factors for dislocation following THA including malposition, sex, a primary diagnosis of THA, surgical approach, preoperative status of the patient, coexistence of neuromuscular disorders, and femoral head size [4–6]. Excluding malposition because accurate component placement was obtained, several factors might have influenced dislocation in the present case. First, a primary diagnosis of ONFH has been considered a risk factor for higher dislocation rate compared with a diagnosis of osteoarthritis (OA) [14]. Patients with ONFH may be characterized by younger age and higher activity than patients with OA. Second, an anterolateral approach may present a smaller risk of dislocation than a posterior approach [6, 15]. Therefore, we traditionally perform THA via an anterolateral approach. Third, as is often pointed out, femoral head size is considered one of the important prosthesis-related factors influencing dislocation. A lager femoral head is considered to allow a better range of motion before impingement and to reduce rate of dislocation [16–19]. In contrast, increased range of motion promotes secondary impingement with a resulting contact between the proximal femur and the pelvic bone. Thus, an appropriate head size is considered to be between 28 mm and 36 mm, and exceeding this diameter is not recommended [5, 16]. Therefore, we selected 28 mm as the size of the femoral head; in addition, a thinner liner with a larger femoral head is a disadvantage for longevity. The targeted CA was also attained in contralateral THA. We instructed our patient to exercise care while raising her legs passively, which may worsen her MG condition. She has been able to walk long distances nearly equivalent to her preoperative level. Dislocation has not recurred.

Considering the above factors together, the main cause of dislocation in this case following THA was probably the coexistence of a neuromuscular disorder, especially the sudden onset of a myasthenic crisis. Although a high dislocation rate (18%) has been reported in a long-term follow-up study of patients with cerebral palsy who underwent THA [20], outcomes after THA with ONFH associated with corticosteroid pulse therapy for MG have not been clear. We consider it important that both physicians and patients recognize the risk of dislocation even after accurate THA, when worsening neuromuscular conditions such as myasthenic crisis are present. Further data are needed to determine the suitability of hemiarthroplasty or dual mobility prosthesis in cases of dislocation. At present, accurate component placement and instructions for preventing dislocation following THA are essential for successful outcomes in patients with MG.

## Conclusions

It should be noted that patients with MG have a potential risk of dislocation due to excessive hip joint laxity during myasthenic crisis, even if accurate component placement in THA is attained and confirmed postoperatively.

## Abbreviations

anti-AChR: Acetylcholine receptor antibody
CA: Combined anteversion
CT: Computed tomography
HHS: Harris Hip Score
MG: Myasthenia gravis
MGFA: Myasthenia Gravis Foundation of America
OA: Osteoarthritis
ONFH: Osteonecrosis of the femoral head
THA: Total hip arthroplasty
UCLA: University of California, Los Angeles
